# High-Field (3.4 T) Electron Paramagnetic Resonance, ^1^H Electron-Nuclear Double Resonance, ESEEM, HYSCORE, and Relaxation Studies of Asphaltene Solubility Fractions of Bitumen for Structural Characterization of Intrinsic Carbon-Centered Radicals

**DOI:** 10.3390/nano12234218

**Published:** 2022-11-27

**Authors:** Marat Gafurov, Yulia Ganeeva, Tatyana Yusupova, Fadis Murzakhanov, Georgy Mamin

**Affiliations:** 1 Institute of Physics, Kazan Federal University, 18 Kremlevskaya Str., 420008 Kazan, Russia; 2 Arbuzov Institute of Organic and Physical Chemistry, FRC Kazan Scientific Center of RAS, 8 Arbuzova Str., 420088 Kazan, Russia

**Keywords:** asphaltenes, bitumen, oil, nanoaggregates, electron paramagnetic resonance (EPR), electron–nuclear double resonance (ENDOR), hyperfine sublevel correlation spectroscopy (HYSCORE), stable free radical

## Abstract

Petroleum asphaltenes are considered the most irritating components of various oil systems, complicating the extraction, transportation, and processing of hydrocarbons. Despite the fact that the paramagnetic properties of asphaltenes and their aggregates have been studied since the 1950s, there is still no clear understanding of the structure of stable paramagnetic centers in petroleum systems. The paper considers the possibilities of various electron paramagnetic resonance (EPR) techniques to study petroleum asphaltenes and their solubility fractions using a carbon-centered stable free radical (FR) as an intrinsic probe. The dilution of asphaltenes with deuterated toluene made it possible to refine the change in the structure at the initial stage of asphaltene disaggregation. From the measurements of samples of bitumen, a planar circumcoronene-like model of FR structure and FR-centered asphaltenes is proposed. The results show that EPR-based approaches can serve as sensitive numerical tools to follow asphaltenes’ structure and their disaggregation.

## 1. Introduction

Because of their ability to precipitate, deposit, and hence interrupt the continuous production of oil from underground reserves, petroleum asphaltenes are considered the most annoying constituent in diverse petroleum systems (petroleum dispersed systems, PDS). Asphaltenes are defined as being soluble in aromatic solvents such as benzene and toluene and insoluble in light alkanes such as n-pentane and n-heptane [1,2]. The phase changes, viscosity, stability, and interfacial properties of PDS are strongly affected by asphaltenes [3,4,5]. It was also stated, for example, that asphaltenes are the most chemically reactive PDS fraction [6]. For this reason, it is necessary to have an understanding of the association of these molecules, their properties, and their stability in PDS.

Currently, there is still ongoing discussion in the literature about the structure, properties, and intra- and intermolecular interactions of asphaltenes and their aggregates. It is known that asphaltenes molecules comprise fused-ring structures, naphthenic rings, and some aliphatic chains, as well as minor amounts of heteroatoms, such as nitrogen, sulfur, and oxygen, and trace metals, such as vanadium and nickel [2,3,4,5,6,7,8,9,10,11]. It is agreed that PDS asphaltenes have similar characteristics, are monomeric, and have a molecular weight of between 200 g/mol and 1500 g/mol [5,12,13]. However, when it comes to the models and mechanisms of asphaltenes aggregates, their stability, their solubility, and even their core organization, the heated debate does not end [2,4,5,14,15,16,17]. This implies that advanced investigations into them call for using various nondestructive experimental approaches.

Asphaltenes contain intrinsic paramagnetic species (paramagnetic centers, PCs) to be studied with electron paramagnetic resonance (EPR) techniques [18,19,20,21,22,23,24,25,26,27,28,29,30,31,32,33,34,35,36,37,38]. Concentrations of PCs in asphaltenes can reach values of 10^22^ spins per gram [13], meaning that every asphaltene molecule can be paramagnetic and that EPR is a suitable (valid) and unique tool for studies on PDS and PDS asphaltenes. EPR does not require additional sample preparation (e.g., dilution), is nondestructive, and allows one to study solid and liquid PDS samples. It is established that PCs concentration directly correlates with the aromaticity factor and C:H ratio but inversely correlates with the solubility of asphaltenes [13].

EPR is sensitive to various electron–nuclear, electron–electron, electron–lattice interactions and, correspondingly, to the nanoscale changes (up to 8 nm) of the local environment of paramagnetic centers. It is shown that PCs can serve as effective probes for PDS to follow the processes of various external treatments, such as having an influence on temperature [20], electromagnetic field [26], or supercritical water [33] and on other types of improved-oil-recovery (IOR) and enhanced-oil-recovery (EOR) methods [4].

The main paramagnetic centers in PDS are the complexes of d-metals (mostly V) and stable carbon free radicals (FR): unpaired electrons delocalized over many conjugated or aromatic chemical bonds. In a series of papers, we have shown that various EPR parameters that are intrinsic to many PDS paramagnetic vanadyl porphyrins (VP, with the skeleton VO^2+^) can serve as excellent probes to study asphaltene aggregation [19,20], transformation [20,33], and deposition [18,19,39]. However, because of the intricacy of the examined PDS and the various types of aggregation, the actual nature and structure of the intrinsic paramagnetic centers, especially FR, remain unknown.

FR is often detected by using standard X-band EPR (microwave frequency ν_MW_ ≈ 9.5 GHz) as a single line (*S* = 1/2) with a peak-to-peak linewidth of ∆Hpp = 0.4–0.8 mT and a *g*-factor between 2.0008 and 2.0081 [29]. Although the intensity of EPR line (concentration of FR), the values of *g*-factor and ∆B_pp_, and the relation between the Lorentz and Gaussian line shapes are specific for every type of petroleum system, the FR EPR signal does not readily reveal information about FR structure.

The availability of pulsed and high-field EPR spectrometers opens new opportunities for PDS analysis, the structural investigation of asphaltenes, and studies on their treatment processes, using intrinsic paramagnetic centers as sensitive probes [18,19,30,31,32,33,34,39,40]. While it is possible that relaxation and spectroscopic parameters of FR derived at various microwave frequencies can provide additional information about the asphaltene structure, this type of investigation is quite rare and does not cover even a small portion of all possible cases, because of the complexity of PDS and the minor number of PDS species studied thus far. For example, in paper [33], an influence of supercritical water (SCW) on FR EPR parameters of Achalchinskoe oil was investigated. It was demonstrated not only that the concentration and line shape of FR was changed but also the electronic relaxation times T_1e_ and T_2e_ of asphaltenes FR were affected during the SCW conversion. Additionally, there is a surprisingly small number of publications reporting on the use of double resonance techniques such as electron–nuclear double resonance (ENDOR) and dynamic nuclear polarization (DNP) in which both EPR (microwave) and nuclear magnetic resonance (NMR, radiofrequency, RF) electromagnetic waves are applied to crude oils and their fractions (see [35,41,42,43,44,45]). The role of FR on aggregation processes and on changes of NMR parameters is undisclosed [10].

The purpose of this work is to investigate the abilities of pulsed EPR (at the W-band range, ν_MW_ ≈ 95 GHz) to characterize FR in asphaltene solubility fractions of bitumen by tracking the changes in spectroscopic and relaxation parameters of FR as well as ^1^H ENDOR spectra. Some EPR results were made publicly available [30,46]. This paper is an extension of our previous work in that it exploits the ENDOR technique by focusing on the FR structure.

## 2. Materials and Methods

The EPR method is based on the resonant (selective) absorption of microwave energy by PCs in an external magnetic field, *B*_0_. The EPR spectra observed in petroleum components are due to the presence of magnetic moment for unpaired electrons and are affected by several types of interactions between the unpaired electron and its environment [38]: (1) the Zeeman interaction between the unpaired electrons and *B*_0_, (2) the spin–orbital interaction, (3) the electron–nuclear interaction, (4) the interaction with other unpaired electrons (spin–spin interaction), (5) and the interaction with the environmental bath (spin–lattice interaction).

The most significant contribution is the Zeeman interaction determined by the expression:

(1) $$h\nu =g\beta B_{0}$$

where *h* is Planck’s constant, *ν* is the microwave frequency, *β* is the Boron magneton, *g* is the spectroscopic splitting factor, and *B_0_* is the value of the induction of the magnetic field. As was already pointed out in Introduction, it was found that the value of the FR *g*-factor, which is close to the *g*-factor of a free electron, depends on the chemical environment of the unpaired electron. Its deviation from the value of 2.0023 depends directly on the content of heteroelements in asphaltenes and has an inverse dependence on the aromaticity factor of asphaltenes [13,21].

The conventional (continuous wave, CW) and pulsed EPR spectra were registered with Bruker ElexSys 580/680 EPR spectrometers. CW EPR experiments were conducted in the X-band (ν_mw_ = 9.6 GHz) and W-band (ν_mw_ = 94 GHz) frequency ranges, with a magnetic sweep field from 5 mT to 1.5 T for the X-band and 3300 mT to 3500 mT for the W-band. The experimental configurations (modulation *A* = 0.1 mT, time of integration τ_int_ = 82 ms, and power of mw-source *P*_mw_ = 100 μW) were tuned to prevent the saturation or distortion of EPR signals.

To get detailed information about the electron–nuclear and spin–lattice interactions, pulsed EPR experiments were conducted. An electron-spin-echo (ESE) signal was detected with the following pulse sequence:

π/2 − τ − π − τ − ESE,(2)

with a π/2 pulse duration of 16–32 ns and time delay τ = 240 ns or longer. Spectra simulations were conducted in the Easyspin package for MatLab [47].

To follow ESE decay and extract the time of transverse (spin–spin, phase–memory) relaxation, T_2_, the same pulse sequence was applied by increasing the time interval τ between the pulses, with a step of 4 ns or longer. To measure the longitudinal (spin–lattice) relaxation time, T_1_, the inversion-recovery three-pulse sequence was used:

π − T_d_ − π/2 − τ − π − τ − ESE, (3)

with a π/2 pulse duration of 16–32 ns and time delay τ = 240 ns or longer.

Electron-spin-echo envelope modulation (ESEEM) was implemented using a well-known two-pulse Hahn sequence: π/2 − τ − π, where the length of π was 32 ns and τ was equal to 200 ns. In this method, the integral intensity of ESE was recorded, depending on the time interval, τ, between two pulses at a fixed magnetic field. This parameter was increased to the required value (before the loss of information on nuclear modulations). To gain the sensitivity and spectroscopic resolution, the smallest possible step, t = 4 ns, was chosen, and the ESE was integrated only at its peak, with an integration time of t = 4 ns. Further spectral analysis of the obtained results involved a Fourier transform using the program OriginPro.

**Figure 1:**
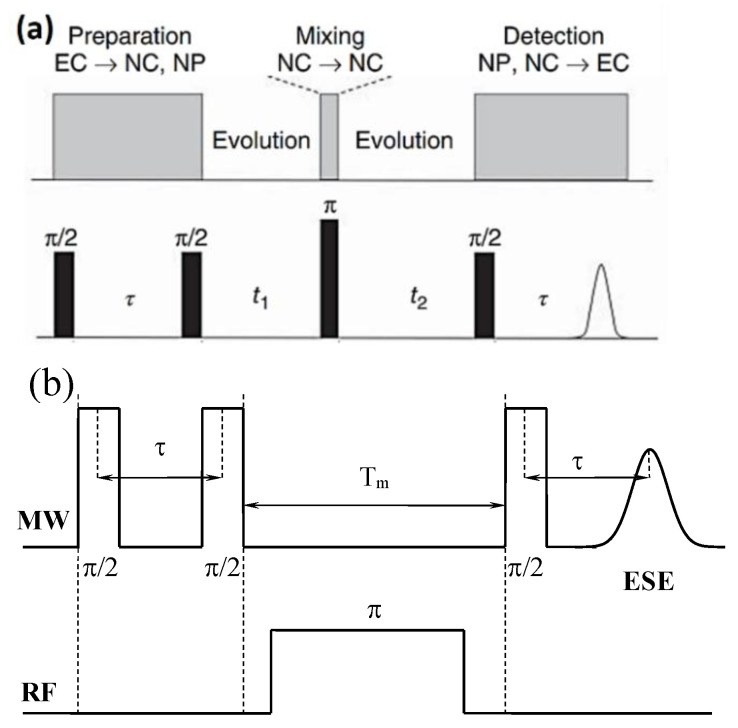
(**a**) Pulse sequence to acquire two-dimensional signals from electron–nuclear interactions (HYSCORE), where NP stands for nuclear polarization, NC stands for nuclear coherences, and EC stands for electron coherences; (**b**) pulse sequence to obtain Mims ENDOR spectra.

In the case of the presence of anisotropic electron–nuclear interactions caused by magnetic nuclei, a modulation of the ESE decay might be observed [40]. It offered an opportunity to identify an FR nuclear environment and estimate the values of their hyperfine interactions with electron spin. A four-pulse sequence, as shown in Figure 1a, was conducted in the form of a stimulated echo sequence, by inverting a 180-degree pulse inserted after the second π/2 pulse. The application of a π pulse led to a mixing of the nuclear frequencies and offered a possibility to apply hyperfine sublevel correlation spectroscopy (HYSCORE) techniques [40].

The ability to detect ESE offered an opportunity to obtain ENDOR spectra by using a Mims pulse sequence with an RF pulse of about 18 μs (the pulse length was optimized for ^1^H nuclei) inserted between the second and third microwave pulses (Figure 1b). RF frequency in our setup could be swept in the range of 1–200 MHz. More details on the pulsed W-band EPR and ENDOR measurements for PDS with the corresponding references were given in papers [18,19].

Asphaltenes were extracted from the Zuzeevsk bitumen (Tatarstan, Russia). Some properties of the initial sample are listed in Table 1.

Asphaltenes (Ainit) were precipitated from the raw material by the addition of 40 mL·g^−1^ of the petroleum ether (b.p. 40–70 °C). Precipitated asphaltenes were washed in a Soxhlet apparatus with petroleum ether until the filtrate became colorless. Then the asphaltenes with a filter were washed out with benzene, which was then evaporated.

The A1 and A2 fractions of asphaltenes were obtained as follows [30]. One gram of Ainit asphaltenes was first completely dissolved with 28 mL toluene. Petroleum ether (b.p. 40–70 °C) was then added in the amount of 52 mL. The resulting solution was kept in a dark place for 24 h and then filtered. Precipitated material was washed in a Soxhlet apparatus with toluene until the solvent became colorless and was dried and weighed until there was no change in mass. Obtained fraction was called A1. Then the petroleum ether was added to the supernatant in the amount of 228 mL and precipitated for 24 h. Using the operations described above, the fresh precipitated material was separated, washed with toluene in a Soxhlet apparatus, and dried. The A2 fraction of asphaltenes was recovered by evaporating the remaining solvent. According to the results of various investigations reviewed in paper [5], subfraction A1 has a high molecular weight and aromaticity factor, has a low H:C atomic ratio, and contains highly condensed aromatic compounds (“island-like”). Subfraction A2 has a high H:C ratio and a relatively low aromaticity factor (“archipelago-like”). The toluene-to-heptane solvent transfer-free energies of steam-cracked tar and petroleum asphaltenes are consistent with the observation that more alkane-soluble subfractions will be dominated by smaller molecules [49,50]. The results of papers [10,51,52,53] suggest that asphaltene polydispersity plays a major role in determining asphaltene precipitation and deposition tendencies observed in different oils.

## 3. Results and Discussion

**Figure 2:**
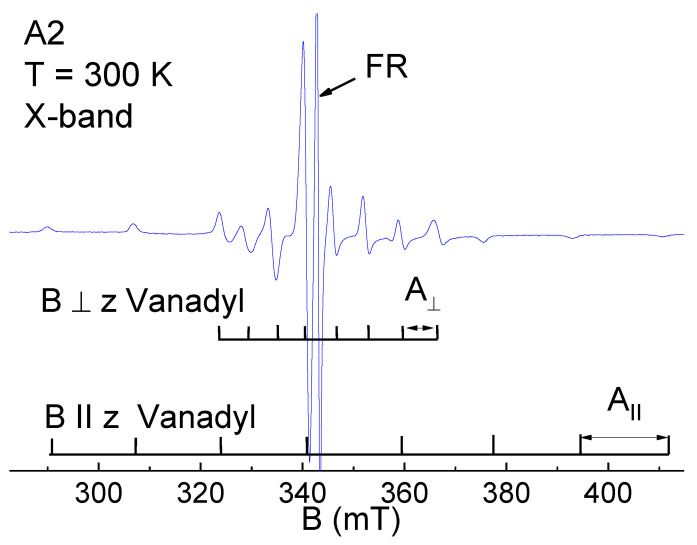
X-band CW EPR for an A2 fraction.

As an example, EPR spectrum for A2 in CW mode is shown in Figure 2. It was due to the presence of two types of PCs. The first one (with a 16-line pattern for the powder spectrum) belonged to the VO^2+^ complex with a *g*-factor of axial symmetry (nuclear spin I = 7/2 for ^51^V nuclei, g_||_ = 1.963, g_⊥_ = 1.985, A_||_ = 468 MHz, A_⊥_ = 150 MHz). A detailed analysis of signal from VO^2+^ complexes was given in our papers [20,44]. Another one was a single line from FR.

A usual way to fit the EPR line shape of FR at X-band is to make it a sum or convolution of Gauss and Lorentz, where Γ_G_ ≈ Γ_L_ (the line width (Γ) for Gaussian (G) and Lorentzian (L) absorption line shapes are connected with the full width at half maximum (FWHM) via Γ_G_ = (2ln2)^−1/2^·FWHM and Γ_L_ = (3)^−1/2^·FWHM (see [45] for details) and the isotropic *g*-factor (g_iso_ ≈ 2.0038(3)). Measurements in the X-band show that the parameters of FR spectra do not depend on fractioning.

**Figure 3:**
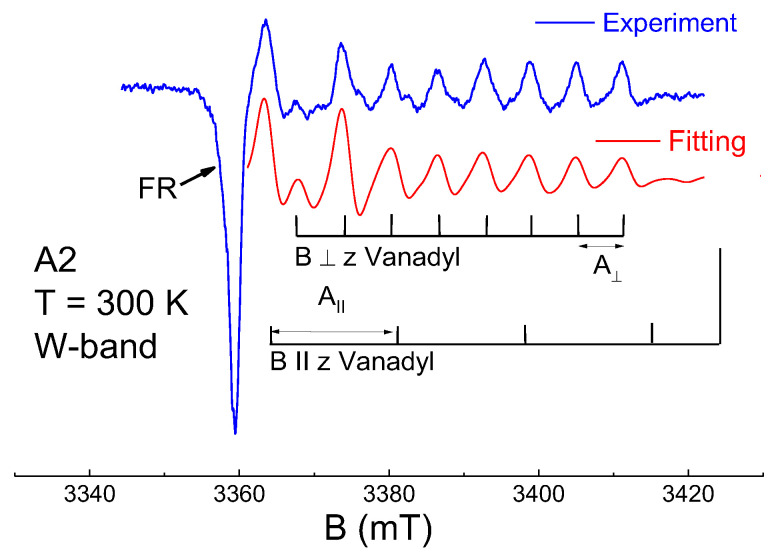
W-band cw EPR for A2 fraction.

**Figure 4:**
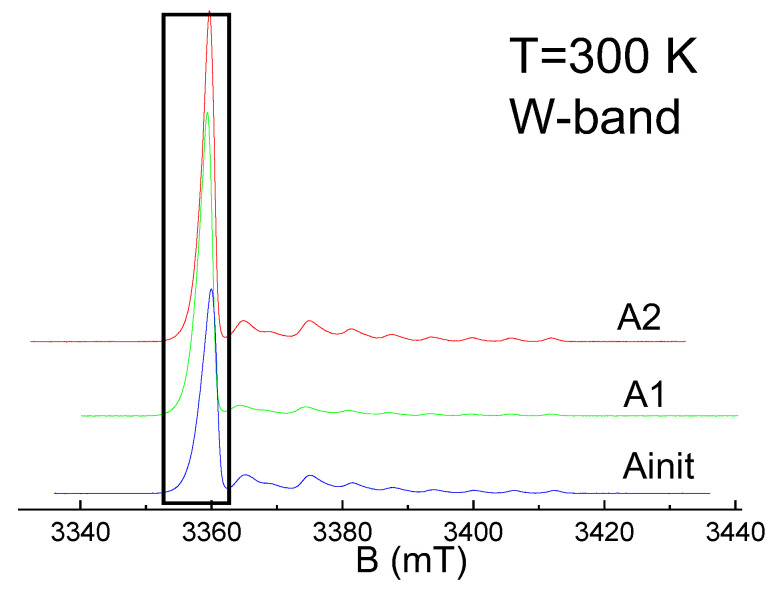
W-band ESE EPR for A1, A2, and Ainit fractions. Region of FR signal is marked (see Figure 5 for details).

**Figure 5:**
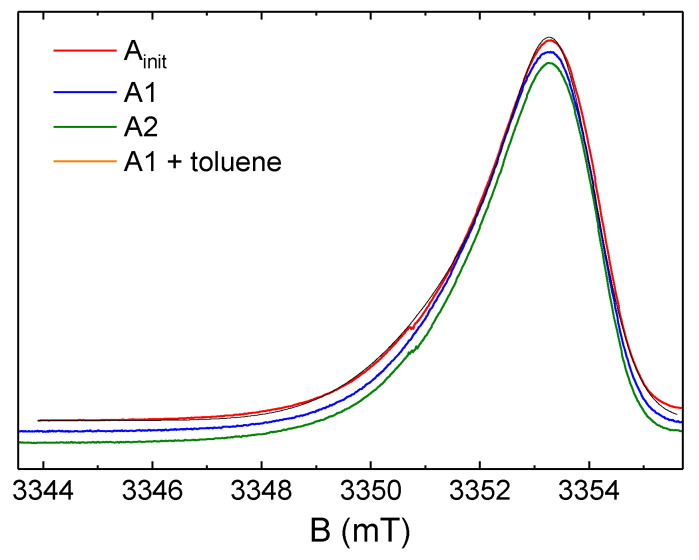
Comparison of the normalized ESE detected W-band EPR (Ainit, A1, A2) registered at T = 300 K and their corresponding fitting as the paramagnetic center of axial symmetry, with g_‖_ ≈ 2.0045 and g_⊥_ ≈ 2.0029. A1 in deuterated toluene (v:v = 1:20) was registered at T= 200 K.

W-band spectra in CW and ESE modes are presented in Figure 3, Figure 4 and Figure 5. As seen in them, in contrast to the X-band, in the W-band EPR, the lines from FR are not symmetric and their line widths become larger. FR signals in the W-band can be ascribed to single paramagnetic center of axial symmetry, with g_‖_ ≈ 2.0045(3) and g_⊥_ ≈ 2.0029(3). As seen in Figure 5, even high-resolute EPR spectrum does not allow obtaining information on the FR structural changes during the asphaltene fractioning or dissolution. This may lead to the incorrect conclusion that the possibilities of EPR are extremely limited. More-elaborated EPR techniques, as will be shown below, should be applied in studies on petroleum systems.

**Figure 6:**
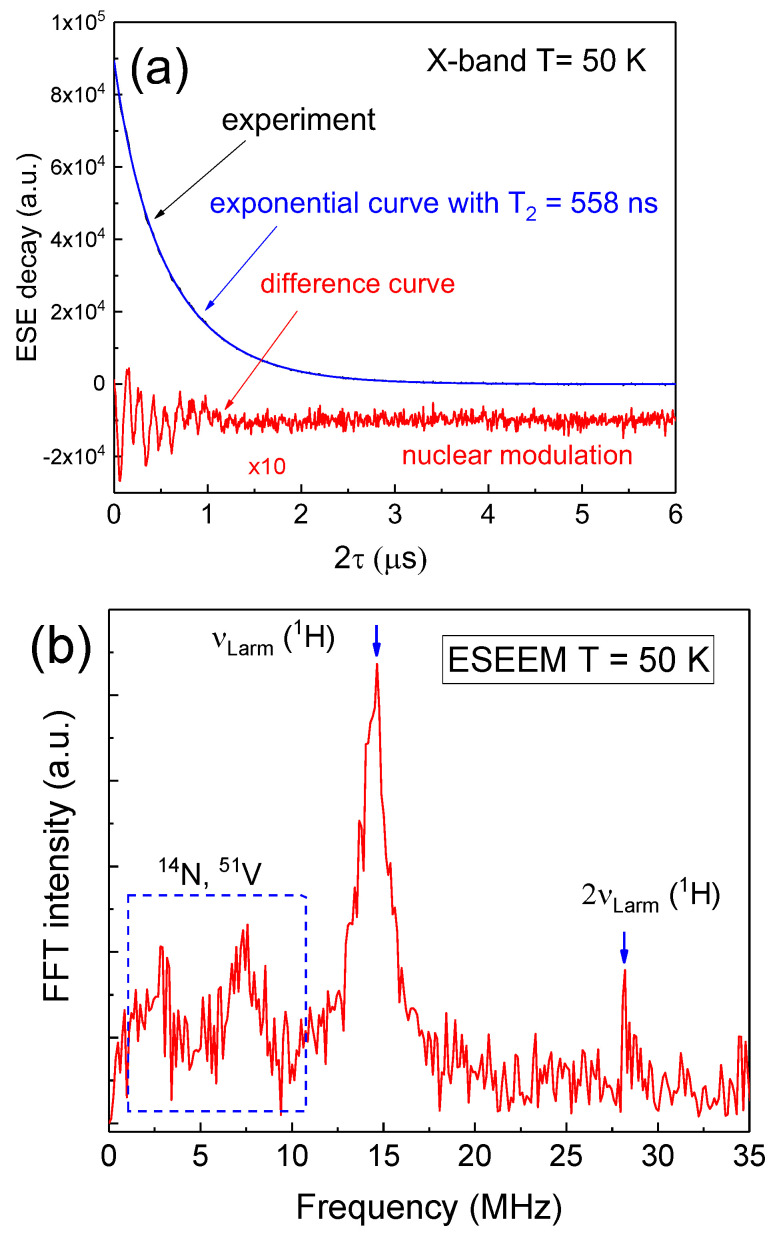
(**a**) FR ESE decay in the X-band for A2 registered at T = 50 K (black); T_2_-curve (blue) and their scaled difference (red); (**b**) fast Fourier transformation from the red curve in Figure 6a. Values corresponding to the Larmor frequencies for ^1^H, ^14^N, and ^15^V are marked (cf Table 2).

The decay of the transverse magnetization (ESE decay, T_2_-curve) in the X-band contains modulations (ESEEM, [54]) were caused by anisotropic electron–nuclear interactions (Figure 6a), although the modulation amplitude (modulation depth) was at least an order of magnitude less than that for the vanadyl complexes (cf with the results in paper [40]). Therefore, the measurements were demonstrated for T = 50 K to increase the signal-to-noise ratio. A Fourier-transformed signal in the frequency domain is shown in Figure 6b. It allows for identifying the nuclear environment of FR. As seen in Table 2, the obtained signals belonged to interactions with hydrogen (^1^H) nuclei in the high-frequency region and unresolved signals from ^14^N, ^51^V, or even ^13^C in the low-field part. In the W-band, the modulation was not registered, although the values of T_2_ in particular did not change with the mw frequency (T_2_ = 400–450 ns for T = 300 K). Similar to the EPR results (Figure 4), ESEEM did not depend on the type of extracted asphaltenes (sub)fraction.

A comprehensive analysis of the mutual impact of FR and VP on various asphaltene (sub)fractions as extracted from the electronic relaxation times measurements was conducted in paper [30]. Because of the spin–spin (FR-VO^2+^) interaction, it may lead to the shortening of FR relaxation times. It was derived from the relaxation data that FR and VO^2+^ are located on the distance of 0.8–1.0 nm and form complexes that can be destroyed by dissolution.

**Figure 7:**
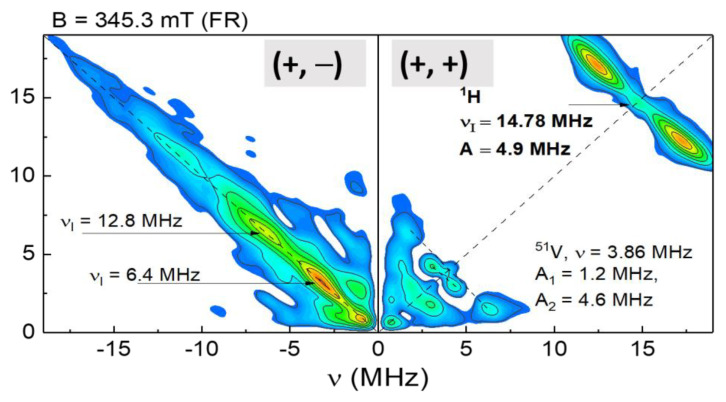
FR HYSCORE for A2.

To obtain more-detailed information on the FR-VP structure, we used HYSCORE. X-band HYSCORE for FR is shown in Figure 7. The left part (quadrant) of the spectrum consists of intense but structureless signals that are located along the diagonal with a wide distribution of hyperfine value, from almost 0 MHz to 15 MHz (with two intense peaks at 6.4 MHz and 12.8 MHz). It was probably due to the wide distribution of distances between FR and other nuclei, for example forming a VP skeleton, specifically ^14^N and ^51^V. Thus, FR, at least with respect to ^14^N and ^51^V, did not have a definite localization in the VP structure. In the right quadrant of Figure 7, it is possible to observe the signal from hydrogen ^1^H with a split of 4.9 MHz. This pattern suggests that the FR has a quite definite structure with respect to hydrogen and that therefore ^1^H-electron hyperfine values might give essential information on the changes in asphaltenes structures. The low-frequency part of the right quadrant can be ascribed to an interaction with ^51^V and ^13^C (see Table 2). In the W-band, HYSCORE experiments were not conducted, because, as was pointed out above, the ESEEM signal was too weak.

**Figure 8:**
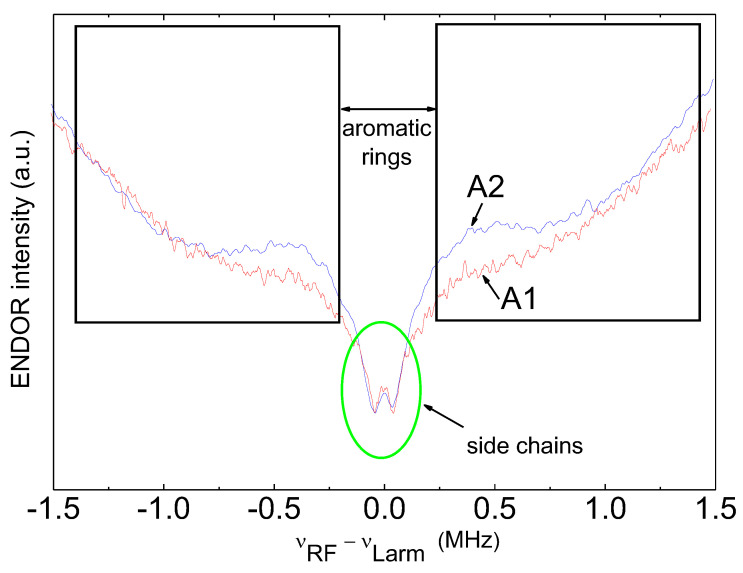
^1^H ENDOR spectra for A1 and A2 fractions.

Now consider how the nuclear environment of paramagnetic centers changed by exploiting the abilities of the W-band ENDOR. The ENDOR spectra can be registered both for the FR and for vanadyl porphyrins. VP ENDOR was discussed in detail in [18,19,21]. In this paper, we investigate FR ENDOR. For that, the duration of the mw pulse was chosen to be 44 ns (Figure 1b); thus, the pulse spectrum excited all FRs but did not affect the VP spectrum. In the FR ENDOR, only an interaction with ^1^H was observed; no interaction with ^13^C, ^51^V, or ^14^N was detected. As seen in Figure 8, the ^1^H ENDOR spectrum is typical for the unoriented (powder) samples.

**Figure 9:**
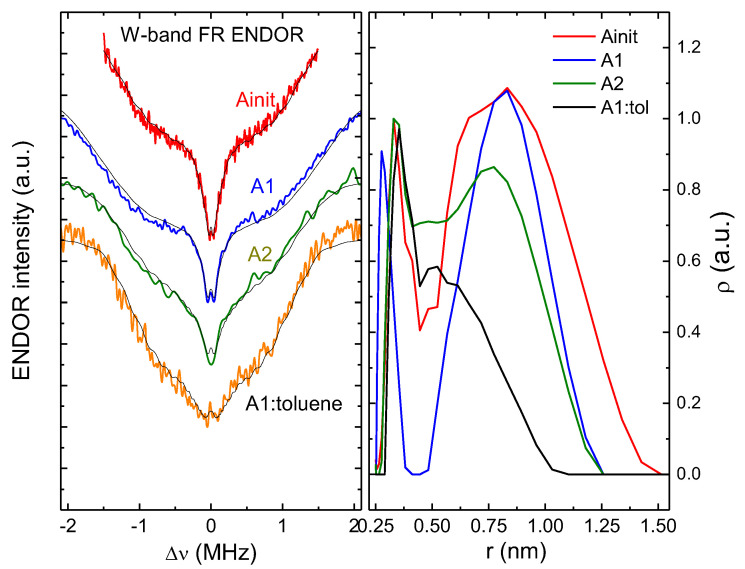
(Left panel) ENDOR spectra for FR in the W-band for the asphaltenes subfractions and A1 dissolved in deuterated toluene (v:v = 1:20); (right panel) distribution function of the contribution of protons from the distance to FR, obtained from the ENDOR spectra.

To describe the obtained ENDOR data, we took a simplified model of the electronic structure of FR. We supposed that the nuclei of the environment affect the electron spin only through the interaction between magnetic dipoles, which is anisotropic in nature. To simplify the calculations, we neglected the admixture of the Pauli (contact) interaction, assuming that such an interaction corresponds to energies above 2 MHz. In this case, it was possible to fit the ENDOR spectrum with good accuracy and determine the contributions to the spectrum of protons located at different distances from the FR (Figure 9). We carried out the spectrum deconvolution procedure with additional conditions for non-negativity and the smoothness of the distribution. When calculating the spectrum, we used convolution with the intensity of the spectrum because of the blind spot, the maximum suppression of which for all spectra was at a frequency of 2.1 MHz. Moreover, 32 amplitude points were used in the distribution of the contribution of protons, depending on the distance. As can be seen in Figure 9, the deconvolution results (thin black lines) quite well describe the experimental spectra.

For all asphaltene (sub)fractions, a peak was observed in the region of 0.75 nm (Figure 9, right panel). It corresponds to the nearest protons in aromatic cores where FR was localized. The initial fraction was characterized by a wide density distribution of interacting protons around FR, up to distance of 1.5 nm. The distance distributions for subfractions A1 and A2 differed in the vicinity of 0.45 nm. For the A1 there were practically no protons in this region, which most likely was due to the prevalence of aromatic cores. In fraction A2, there were a number of protons at this distance because of either alkane chains or the boundaries of the aromatic rings. When dissolved, both fractions showed fewer protons at a distance of 1.25 nm than the initial asphaltenes fraction. This might be attributed to the “washing out” of hydrogen-enriched compounds from asphaltenes.

It was quite notable that transformations of ^1^H ENDOR were observable also with the dissolution of the A1 fraction, which is supposed to be poorly soluble in toluene. A1 was subjected to partial dissociation by dissolving it in deuterated toluene in proportions of 1:5 and 1:20 by volume. Of course, this degree of dilution was not enough for the complete destruction of the asphaltene aggregates [4,5], but as seen in our results, ^1^H ENDOR can serve as a sensitive tool to follow the partial destruction of asphaltene aggregates, along with the electronic relaxation times (cf with [26,30]).

**Figure 10:**
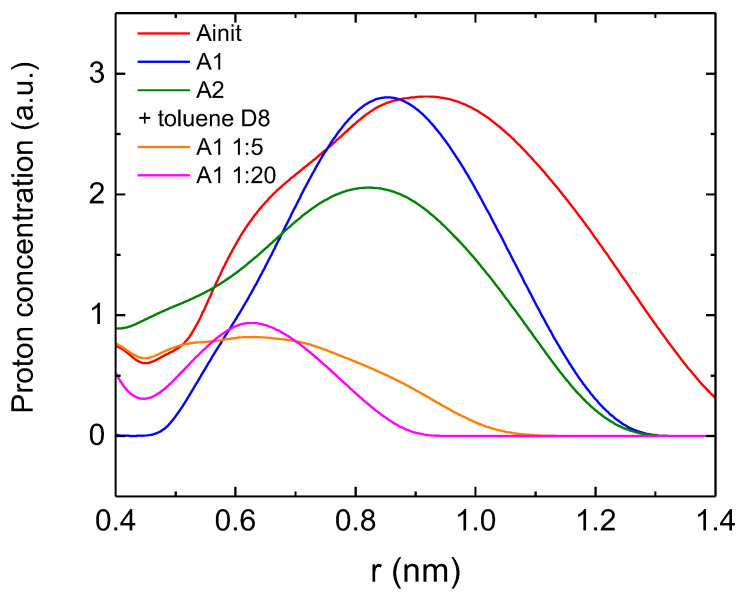
Distribution of proton concentration depending on the distance from the localization site of FR.

To determine the type of distribution of the concentration of hydrogen nuclei, depending on the distance from the paramagnetic center, it was necessary to take into account the decrease in the intensity of the ENDOR signal over distance. For the decrease in the intensity of the ENDOR signal as r^−3^, the calculated proton concentration distribution is shown in Figure 10.

In Figure 10, it can be seen that during the partial dissociation of asphaltene aggregates, protons located at a distance of 0.9 nm were replaced by solvent molecules (group 1), and only protons located on the distance of 0.7 nm remained (group 2). Moreover, at a dilution of asphaltenes in ratio of 1:20, the proton concentration peak became narrower, and the dip around *r* = 0.45 nm became deeper. Unfortunately, the further dissolution of asphaltenes in toluene led to a sharp suppression of the ENDOR signal, hindering further reliable measurements. It can be concluded that the second group of protons belonged to the asphaltene complex itself, whereas the first group belonged to other molecules, ones that are weakly bound to each other. Probably, this is because A1-type molecules stacked with each other (which prevents the protons from approaching FR), whereas A2-type molecules did not or did so to a lesser extent. When asphaltenes were slightly diluted with deuterated toluene, the molecules partially dissociate in the plane and the layers were shifted relative to each other; therefore, protons appeared at a distance of 0.45 nm. Further dilution led to further separation between the layers and a decrease in the number of protons at a distance of 0.45 nm and 0.7 nm.

## 4. Conclusions

Based on the described experiments and calculations, this paper constructed a hypothetical model of a near environment for an FR-centered structure in asphaltenes (sub)fractions and peculiarities of various EPR approaches to characterize asphaltenes.

1. The nearest FR environments in asphaltenes were similar to each other: EPR spectra either in X- or W-band were the same for the (sub)fractions under study.
2. The nearest FR environment in all studied asphaltenes (sub)fractions was planar. One can suggest either a porphyrin-like structure, such as as for paramagnetic vanadyl complexes [13,19,28,35], or a circumcoronene-like structure [55].
3. The wide distribution of distances between FR and other magnetic nuclei, such as ^14^N and ^51^V, as seen in HYSCORE results and low-modulation amplitudes in ESEEM, suggested that ^14^N or ^51^V were not obligatory heteroatoms for the nearest environment of FRs.
4. From ^1^H ENDOR, it followed that for an FR of asphaltenes, the highest proton concentrations were observed at distances of 0.8–1.0 nm from FR core. This value grew in a row: A2→ A1→ A_init_. It follows that fraction A1 consisted of the most symmetrical and homogeneous molecular complexes presumably stacked (which prevents the protons from approaching FR core) on top of one another.
5. ^1^H ENDOR can serve as a sensitive tool to follow the partial destruction of asphaltene aggregates, such as by dissolution. By dissolving in toluene, the peak of the highest proton concentrations shifted to the value of <0.7 nm.

## Figures and Tables

**Table 1:** Content of V, Ni, S [48], and asphaltenes fractions in the sample of the oxidized bitumen.

Vanadium Content (mg/kg)	Nickel Content (mg/kg)	Sulfur Content (Mass. %)	Asphaltene Content, (Mass. %)	Amount of Extracted Asphaltene Fractions (Mass. %)		Percentage of Condensed (Aromatic and Naphthenic) Structures by Thermal Analysis (%)	
				A1	A2	A1	A2
390	85	4.2	34	83	15	61	43

**Table 2:** Frequencies of the experimental obtained (exp.) signals (Figure 5) and calculated (calc.) values of the Larmor frequencies for certain nuclei in the X-band for B = 345.3 mT.

	^1^H, ν_Larm_ (MHz)		^14^N, ν_Larm_ (MHz)		^51^V, ν_Larm_ (MHz)		^13^C, ν_Larm_ (MHz)
	Exp.	Calc.	Exp.	Calc.	Exp.	Calc.	Calc.
FR (B = 345.3 mT)	14.78	14.7	--	1.063	3.86	3.87	3.7

## Data Availability

Data are available upon request from the authors.
